# Observing Single Enzyme Molecules Interconvert between Activity States upon Heating

**DOI:** 10.1371/journal.pone.0086224

**Published:** 2014-01-21

**Authors:** Marcin J. Rojek, David R. Walt

**Affiliations:** Department of Chemistry, Tufts University, Medford, Massachusetts, United States of America; Universidad de Granada, Spain

## Abstract

In this paper, we demonstrate that single enzyme molecules of β-galactosidase interconvert between different activity states upon exposure to short pulses of heat. We show that these changes in activity are the result of different enzyme conformations. Hundreds of single β-galactosidase molecules are trapped in femtoliter reaction chambers and the individual enzymes are subjected to short heating pulses. When heating pulses are introduced into the system, the enzyme molecules switch between different activity states. Furthermore, we observe that the changes in activity are random and do not correlate with the enzyme's original activity. This study demonstrates that different stable conformations play an important role in the static heterogeneity reported previously, resulting in distinct long-lived activity states of enzyme molecules in a population.

## Introduction

Enzymes exhibit both fast changes and long-lived differences in activity [1]–[7]. Fast changes give rise to what is called “dynamic heterogeneity” in which relatively low energy barriers (compared to the Boltzmann energy (k_B_T)) enable rapid interconversion between different conformations at ambient temperature [8]. These dynamic changes are relatively fast and are the result of a wide range of internal motions, such as fluctuations within a group of atoms, within sub-domains, or within the entire protein [9]–[11]. Previous experiments indicate that enzymes can also exhibit long-lived activity differences at room temperature resulting in a distribution of activities between ostensibly identical molecules, termed “static heterogeneity” [2], [3], [12]. This static heterogeneity arises either from different conformations of the enzyme and/or different primary sequences between molecules, with the latter possibility resulting from errors in transcription and translation [11], [13]–[15]. When a nascent protein folds, it is possible that each molecule becomes trapped in a local energy minimum and many different local minima, i.e. different conformations, may be populated with each conformation exhibiting a different activity. Moreover, if different enzyme molecules differ in their primary sequence, they could also exhibit a distribution of activities [16], [17]. In this paper, we demonstrate that static heterogeneity of an enzyme population is caused by the existence of many different yet stable conformations.

## Results and Discussion

**Figure 1:**
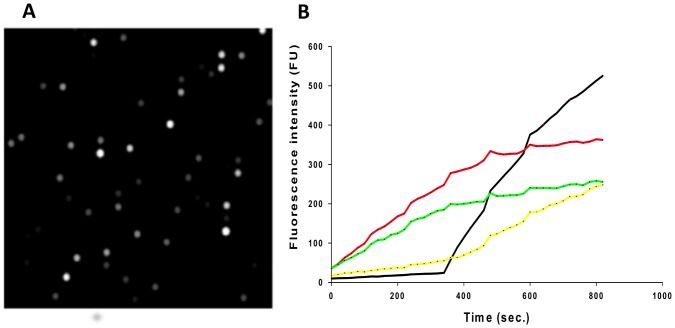
Single enzymes trapped in wells and raw intensities of the trajectories. (A) Image of fiber bundle with trapped active enzymes. Each enzyme generates a high local concentration of fluorescent resorufin. (B) Raw fluorescence intensities of the trajectories of individual β-galactosidase molecules.

We developed a platform to study changes in the kinetics of single non-immobilized enzyme molecules by introducing short heating pulses to the system (Figure S1A in File S1). The assay consists of an array of surface-passivated sealed microwells containing a single active enzyme and a high concentration of the fluorogenic substrate resorufin β-D-galactopyranoside (RBG) (Figure 1A). Upon enzymatic reaction, RBG is cleaved and the fluorescent product resorufin is produced and builds up in the microwells (Figure 1B). The surface modification minimizes any interaction of the trapped enzyme with the surface of the fiber wells. (Figure S2 in File S1) In previous papers, we have described the heterogeneity observed in the activities of individual enzymes, and definitively demonstrated that different initial activities are not caused by surface interactions [18].

There are two possible reasons why the initial activities of the enzymes exhibit such heterogeneity. First, the enzyme molecules may possess different conformations, and consequently different activities, even with the same primary sequence. A second possibility is that the enzymes vary in primary sequence due to errors in transcription and translation. The commercially available β-galactosidase used in this experiment (Sigma Aldrich) is expressed in *E. coli* and therefore it is expected that both the transcription and translation processes are not error-free. Transcription errors (such as polymerase slippage, nucleotide misincorporation) and translation errors (such as amino acid misincorporation, frame-shifts, tRNA misacylation etc.) account for 14% and 29% respectively of the protein population having one or more errors [13]. The probability of a β-galactosidase tetramer molecule (465 kDa) existing without any errors is only 25% [19]. The rest of the population might contain one or more errors in primary sequence. These changes in primary structure might have an influence on the conformational dynamics or energetics, resulting in an altered activity of individual enzymes.

**Figure 2:**
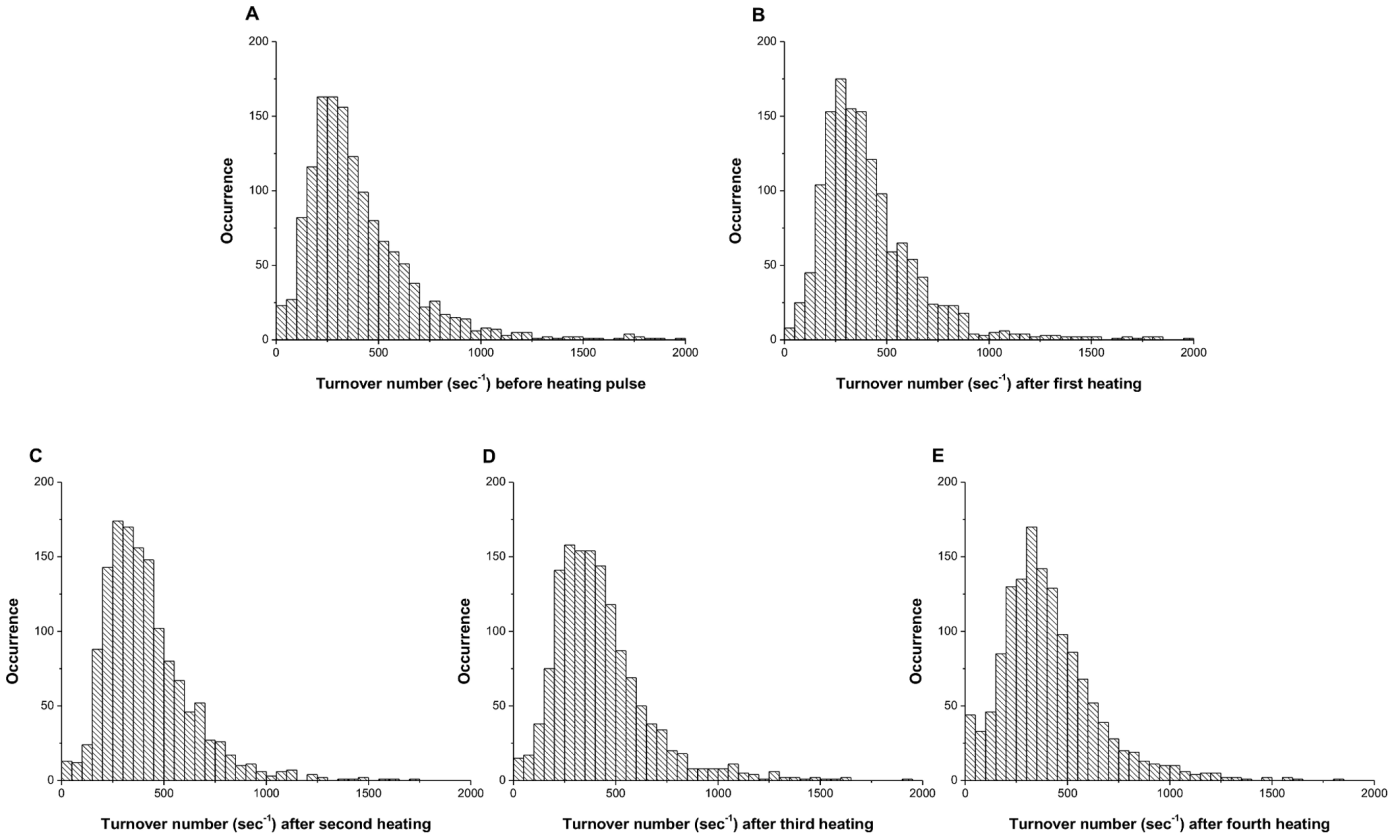
Histograms of activity distribution for single β-galactosidase molecules after each heating cycle. (A) Activity distribution of single β-galactosidase molecules before heating. The mean turnover number for the population of single β-galactosidase molecules of the enzyme population is calculated as 420 sec^−1^. (B,C,D,E) Activity distribution of single enzyme molecules after each sequential heating pulse. The mean turnovers numbers are 426 sec^−1^, 418 sec^−1^, 427 sec^−1^ and 411 sec^−1^ respectively. The bin size for all the histograms is 50 sec^−1^. Measurements were made at 20°C.

**Figure 3:**
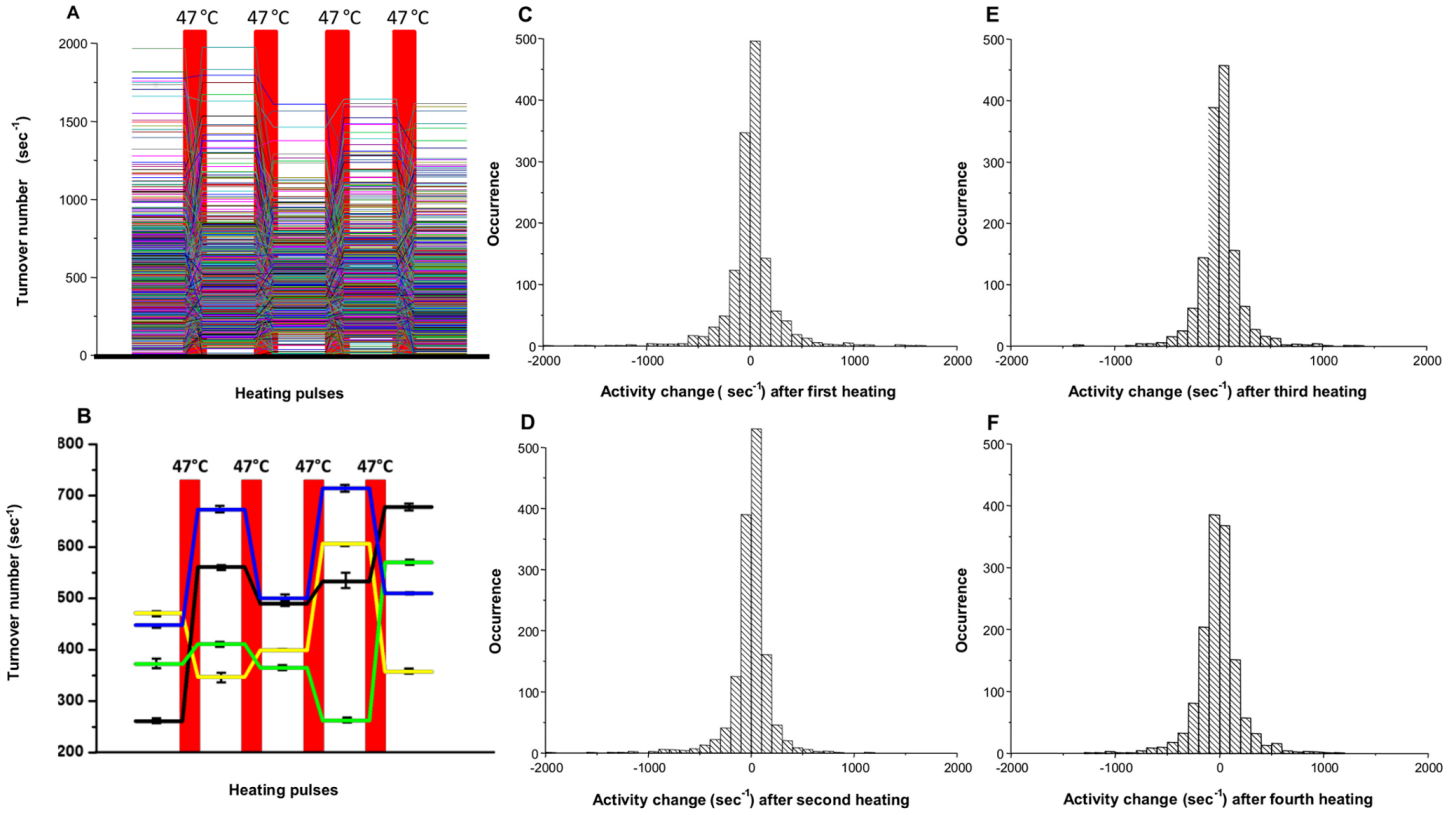
Activity traces and changes in individual β-galactosidase molecules between heat pulses. (A) Activity traces of all the individual β-galactosidase molecules taken from a single experiment. Six images were taken between each heating pulse and the activity was obtained (R^2^ = 0.9 or greater). (B) Only four individual enzyme activity traces are shown to make it clear how the activities change after each heating pulse. The error bars correspond to linear fitting of data between heating pulses. (C, D, E, F) Histogram of the activity changes resulting from heat pulses. Negative values correspond to a decrease in the activity and positive values correspond to an increase in the activity after a heat pulse. The average changes in activity are 4 sec^−1^, -10 sec^−1^, 9 sec^−1^, and -15 sec^−1^ respectively for C, D, E and F.

**Figure 4:**
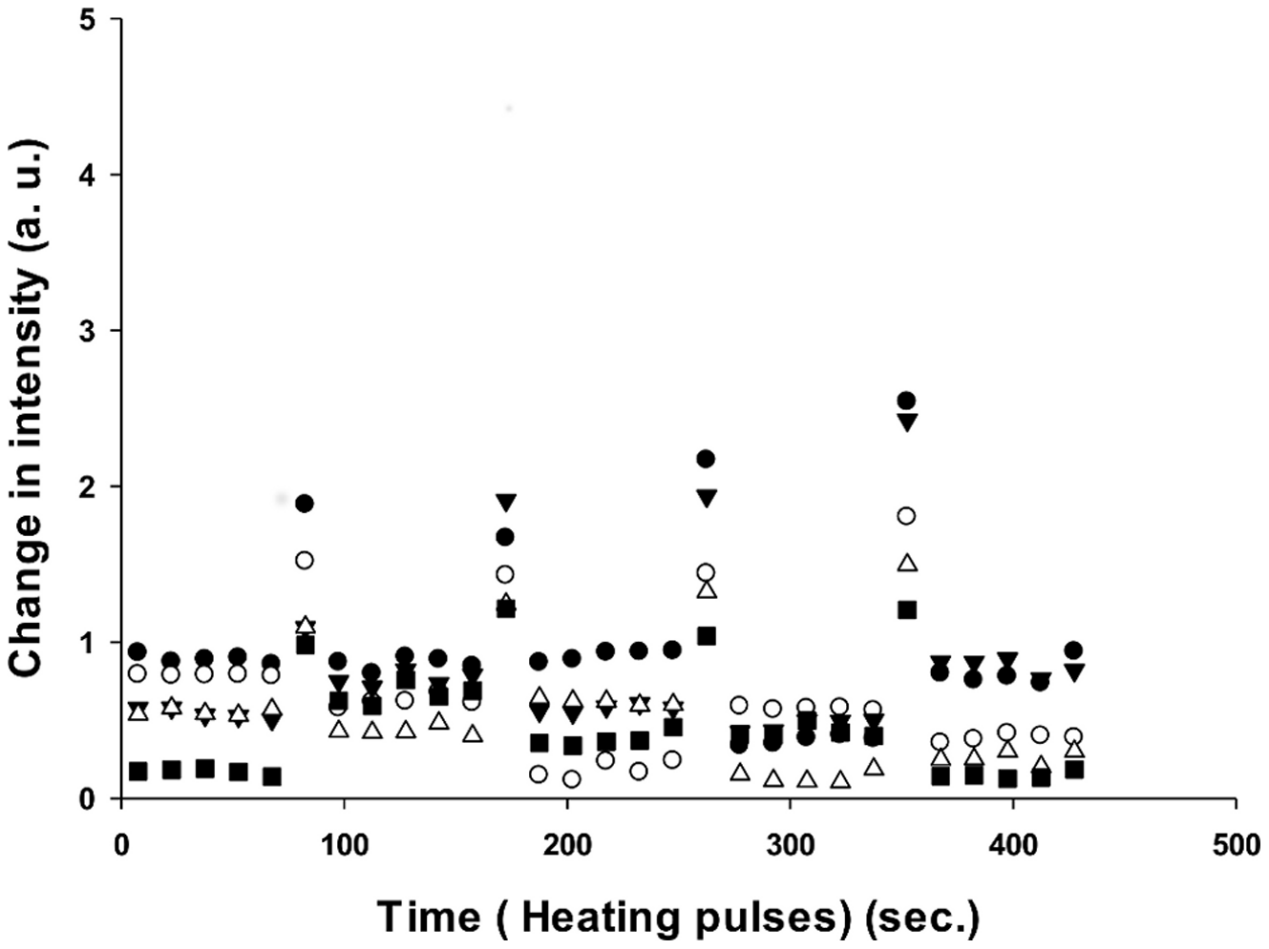
The first derivative plot of raw intensities of individual molecules with respect to time. The activities of the enzymes are relatively stable between the heating pulses. The sudden increases of the activities observed in the graph are due to the heating pulse when the solution did not have time to reach room temperature. Those frames are excluded from the analysis.

**Figure 5:**
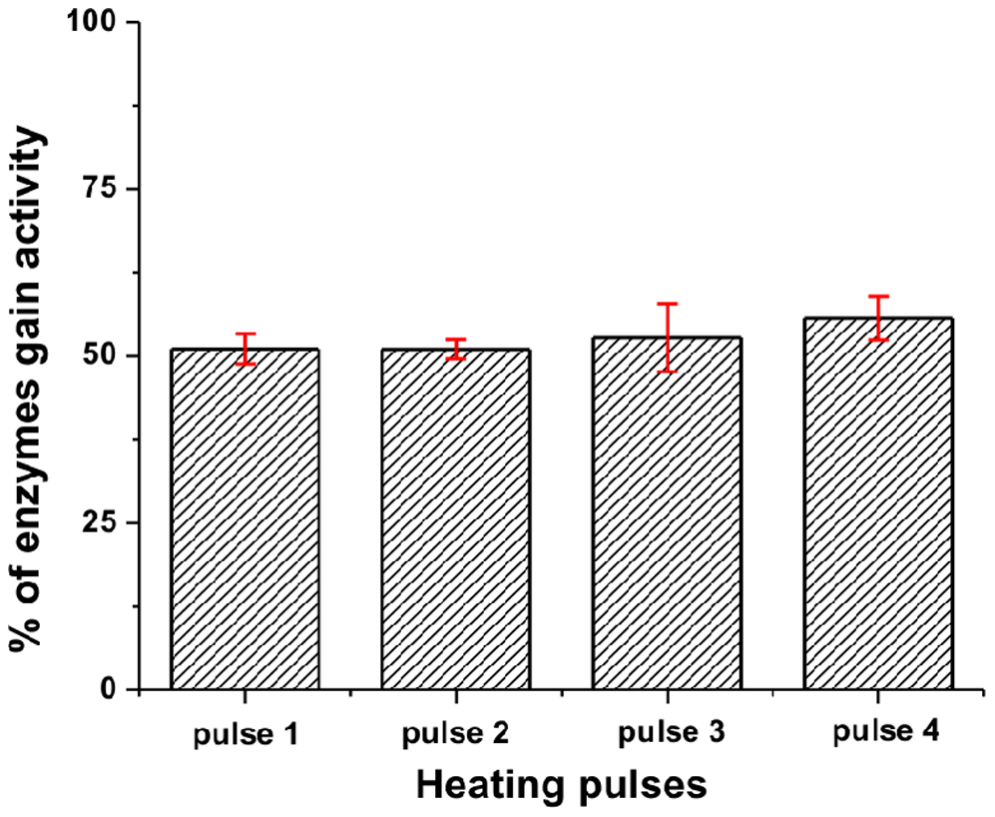
Bar graph representing the percentage of enzyme population gaining activity after each heating pulse. The error bars represent variations from four different experiments.

**Figure 6:**
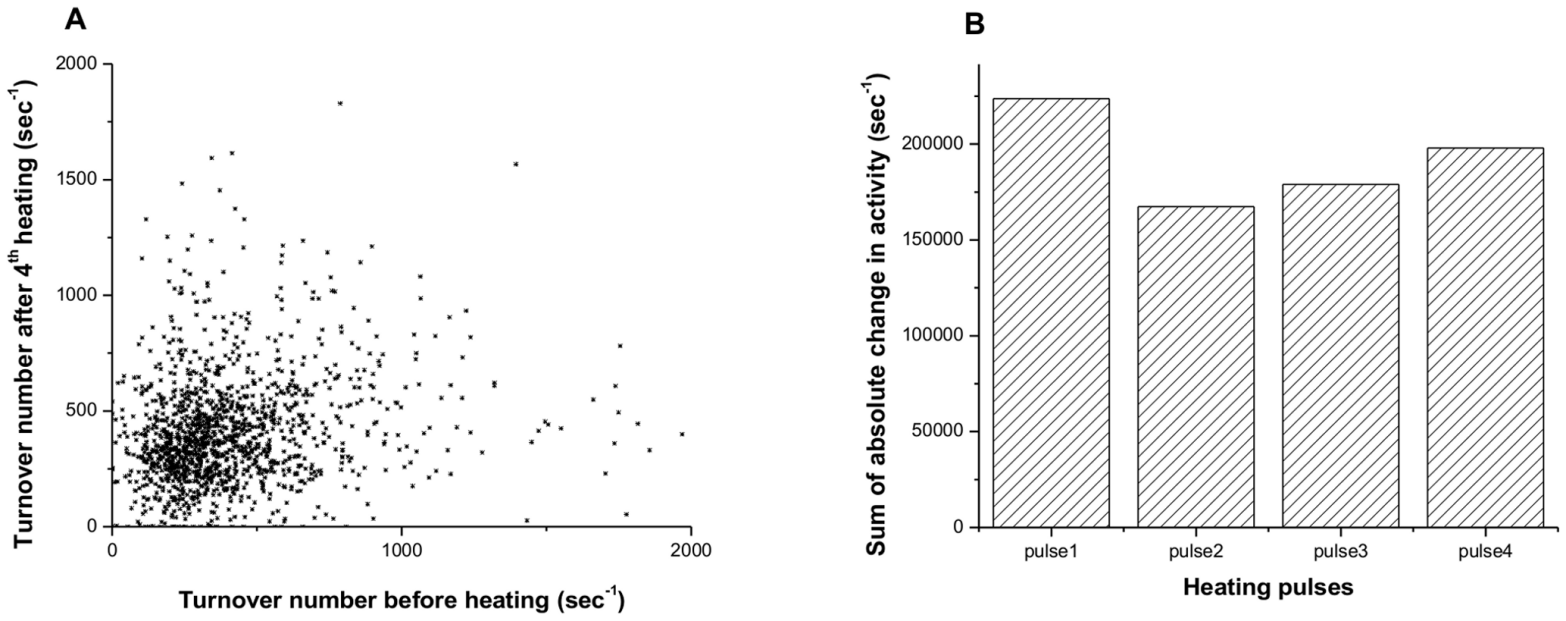
Observations of enzyme activities before and after heating. (A) Plot of the turnover number of enzymes before heating vs. turnover number after the fourth heating. No correlation between activities was observed. (B) Bar graph of sum of absolute activity changes for all enzymes after each heating pulse. No dramatic change is observed between different heating pulses.

**Figure 7:**
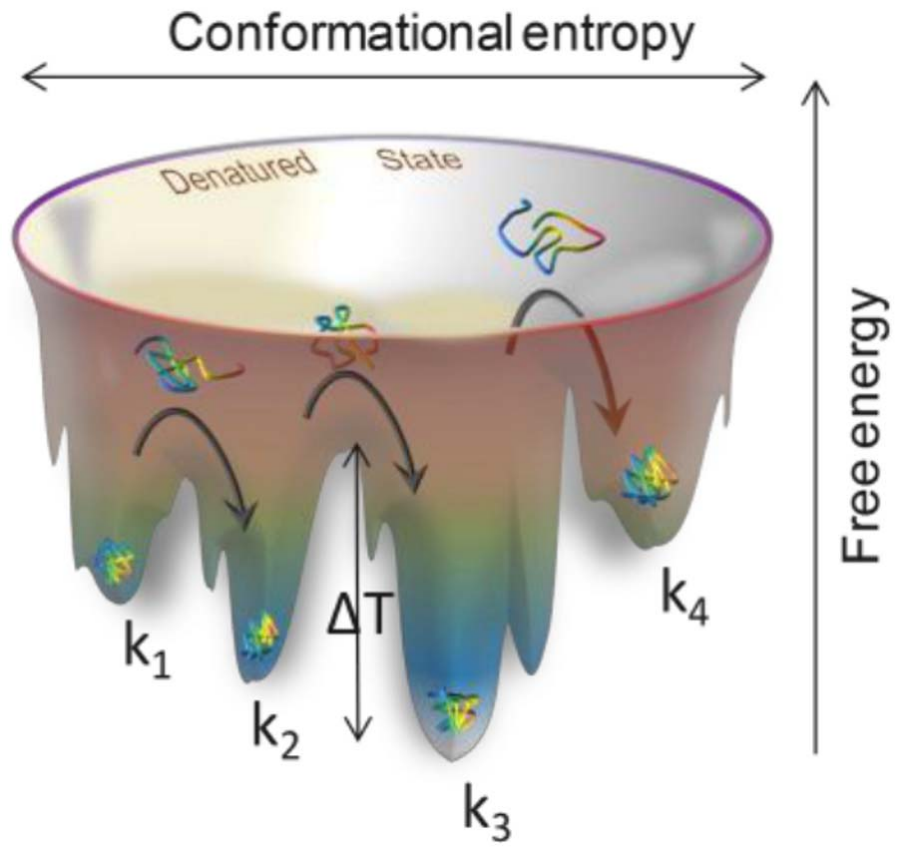
Depiction of the energy landscape. An enzyme with a characteristic rate k_1_ is trapped in a local minimum. When a heat pulse is introduced (ΔT), the enzyme changes its conformation and relaxes into a different minimum. The new conformation exhibits a different rate (k_2_). After sequential pulses, the enzyme can adopt different conformations leading to different rates (k_3_, k_4_).

In our experimental design, we employed a heating stage to introduce thermal pulses to perturb the conformation of the enzyme molecules. In our system, we observe the activity changes resulting from conformational changes, however we cannot observe individual protein conformations directly. If conformational differences are the cause of static heterogeneity, we would expect that heat pulses would convert some of the molecules into different conformations and we would observe changes in activity from the initial activity state after cooling. On the other hand, if sequence differences are the primary determinant of activity, then heating would not be expected to change the activity. After obtaining the initial activity of the enzyme molecules, five short heating pulses were introduced (Figure S1E in File S1) and the activities were measured after each pulse (Table S1 in File S2). When a population of enzymes was exposed to pulses of heat, the activity distributions of the entire population of β-galactosidase molecules narrows slightly after the first two heating pulses. After the second heating pulse, the activity distributions of the populations do not change. Importantly, the average activities of the population after each heating pulse were similar to the average activities of the enzymes prior to heating (Figure 2, A–E). When the activities of individual β-galactosidase enzymes were studied, the turnover rates of individual enzymes changed randomly upon introducing a heat pulse and then remained constant during the period between heat pulses. (Figure 3A, B, Figure 4). Enzyme activity was stable (R^2^>0.9) during the 120 sec (6 frames) between the heat pulses (Table S2 in File S2). After each heating cycle, the activity of individual enzyme molecules changed randomly from its prior activity, with approximately 50% of the population gaining activity and 50% losing activity (Figure 3, C–F, Figure 5, Figure 6B). Considering the error associated with linear fitting, a maximum of 12% of the enzyme population may have overlapping activity values after each pulse. There are several possible results that may be expected to occur upon heating an enzyme. First, if the activation barrier is too high for the molecule to convert to a different local minimum, the enzyme will fall back into the same local minimum, maintain its original conformation, and there will be no change in the reaction rate after heating. Second, if the enzyme overcomes the energy barrier, it may convert to a new stable conformation (Figure 7). The new conformation may have either a faster or slower reaction rate. The changes in activity could also be a result of the tetramer-dimer-monomer equilibrium. At the single molecule level, however, the tetramer-dimer-monomer equilibrium is shifted to the monomer form due to the low concentration of monomer in the microwells such that if the tetramer dissociates, we would expect an irreversible loss in enzyme activity—a phenomenon we observe for a small number of enzyme molecules in these experiments. Narrowing of the distribution after the first heating pulse may be caused by kinetic traps that have slightly higher energy barriers and are only accessible when the nascent protein folds; thus some enzymes cannot return to their previous states upon re-heating at 47°C. No correlation between the activities of enzymes before and after heating was observed, suggesting that enzymes have no ‘memory’ of any previous conformations before the heating pulse was introduced. (Figure 6A) The change in activity of each enzyme was random as well, i.e. it did not correlate with its initial activity. Each enzyme exhibits an equal probability of either gaining or losing activity through all heating periods (Figure 2C–F, Figure 5). As temperature increases, a protein molecule is expected to gain enough energy to overcome an energy barrier and convert to a new conformation that has a different activity. If conformational differences between different enzyme molecules are the cause of the broad activity distribution, then the activities of the individual molecules should redistribute after each heating pulse, which is what we observe (Figure 2A, B). On the other hand, if sequence differences are the sole basis for the activity distribution, then upon heating, each enzyme molecule should revert to its original activity. Our results clearly show that virtually all the enzyme molecules redistribute their activity when heated, demonstrating the importance of conformations in static heterogeneity. It is important to note that both conformation *and* sequence may contribute to the activity distribution because enzyme molecules with different sequences will also interconvert between conformations upon heating. Moreover, enzymes with different primary sequences may display varying kinetic responses upon heating (Figure 3A, B).

In addition to the observed change in activity due to conformational changes, denaturation of some enzyme molecules occurred, which is likely the reason for some loss in activity when activity is measured in bulk solution [20]–[23]. These enzymes are irreversibly denatured and contribute to the decrease in average activity over time. The denaturation temperature of β-galactosidase was calculated to be 55°C by CD experiments and confirmed by kinetic measurements (Figure S3A, B in File S1), and agrees well with denaturation temperatures reported in the literature [15], [24]–[26]. In the present experiments, denaturation of the enzymes was minimized by heating only to 47°C, which is below this denaturation temperature. Enzymes that did not show any activity after three consecutive heating pulses were considered denatured and were not included in the analysis. However, enzymes that lost activity after a heat pulse and gained activity in a subsequent heat pulse were not considered to have denatured. An additional fifth heat pulse was used to determine if the enzyme was denatured. When sufficient energy is introduced, an active enzyme (A) should denature and lose activity (D) based on a denaturation rate k_d_ (Eq.1) and should follow first order reaction kinetics with respect to the heat pulses. (Figure S4 in File S1) The rate of denaturation, (k_d_), is calculated using Eq.2, where the number of active molecules, (N), changes with respect to the heat pulse number, (Hp),
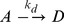
(Eq. 1)

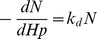
(Eq. 2)and is equal to 0.6% of the population per heating pulse. The total number of enzymes that denatured during the experiment was minimal. There are three major denaturation pathways: aggregation, dissociation, and irreversible conformational changes. Since the enzyme is trapped inside the well, aggregation is ruled out as a possibility. We performed a native gel bulk analysis of the unheated and heated enzyme and only observed bands corresponding to the tetramer for the unheated enzyme. On the other hand, the heated enzyme showed bands corresponding to the monomer as well as the tetramer. (Figure S5 in File S1) This analysis shows that dissociation of the enzyme into monomers occurs under our experimental conditions but reassociation would be virtually impossible as the collision frequency in the microwells is extremely low due to the very low concentration of the four monomers. Finally, the third possible denaturation pathway, irreversible conformation changes, has not been ruled out as a possibility but we do not include any enzyme molecules that exhibit irreversible denaturation in the analysis. It is also plausible that a single enzyme molecule's activity is below the detection limit of 5 sec^−1^, which is only 1% of the average activity for β-galactosidase. No correlation between a particular enzyme activity and its rate of denaturation was observed.

## Materials and Methods

### Materials and Bulk Solutions Preparation

Lyophilized β-galactosidase (Grade VIII) from Escherichia coli was purchased from Sigma-Aldrich, St.Louis, MO. β-galactosidase (1.3 mg) was dissolved in 1.4 mL 1∶1 solution of autoclaved 1×PBS/1 mM MgCl_2_ and glycerol to a final enzyme concentration of 2 µM. Aliquots of 10 µL were stored at −80°C freezer. The 2 µL of enzyme stock solution was diluted in 1×PBS/MgCl_2_ buffer to 360 pM immediately before analysis. Resorufin β-D-galactopyranoside (RBG) and resorufin were purchased from Invitrogen, Carlsbad, CA. Resorufin β-D-galactopyranoside was dissolved in DMSO to a concentration of 100 mM and aliquots of 5 µL were stored at −20°C and diluted to 10 mM in DMSO before each experiment. Ten mM RBG was further diluted in 1×PBS/MgCl_2_ buffer to a final concentration of 100 µM. 396 µL of 100 µM RBG substrate was mixed with 4 µL of 360 pM enzyme giving the working solution of 3.6 pM β-galactosidase. 10× PBS buffer (pH 7.4) was purchased from Ambion, Foster City, CA.

### Bulk experiments

Thermal stability experiments of the RBG substrate and the resorufin product were performed on an Infinite M200 microtiter plate reader (Tecan AG, Mannedorf, Switzerland). The RBG substrate (100 µM) was exposed to different temperatures (40°C, 45°C and 50°C) for 1 min and cooled to room temperature. Each sample was used for an enzymatic assay and compared to an unheated RBG enzymatic assay. The average turnover rate of the enzyme was calculated from these experiments. Both heated and unheated substrates were used, with no difference in enzyme activity observed, indicating that the substrate did not degrade substantially by heating. (Figure S6 in File S1) A similar experiment was performed on resorufin where 100 µM concentrated product was heated at different temperatures (40°C, 45°C and 50°C) for 1 min and cooled to room temperature. The fluorescence intensity (Ex. 558 nm/Em. 590 nm) was recorded over 10 min and compared with unheated product and no difference in fluorescence intensity was observed. In addition, the product's stability was studied on the microscope stage where 46 fL of 10 µM product was trapped in the microchambers. The fluorescence intensities were measured and no significant difference in fluorescence intensity was observed before and after heating pulses at 47°C and 55°C. (Figure S7 in File S1) Activities of the enzyme with respect to temperature changes were measured for the system. The heated stage was equilibrated to a specific temperature and single molecule experiments were performed as previously described. For each temperature, the activities of 1000 molecules were obtained and averaged and then plotted against the temperature. The optimum temperature of the enzyme for maximum activity was obtained and found to be 55°C. Beyond this temperature enzyme activities decreased. (Figure S3B in File S1) In addition, the conformational stability of the enzyme was studied using circular dichroism, JASCO J-720 spectropolarimeter, with a peltier pump attached. The readings were performed at 222 nm with a temperature ramp of 1°C per min. (Figure S3A in File S1)

### Microarray preparation

Custom made optical fiber bundles of ∼50,000 4.5 µm diameter optical fibers purchased from Schott North America Inc. Elmsford, NY were cut to lengths of 4.5 cm. Both sides of the bundles were polished using a fiber polisher (Allied High Tech Products Inc, Rancho Dominguez, CA). One end of the fiber bundle was etched in 0.025 M HCl for 115 sec. The differential rate of etching between the core and the cladding results in an array of 2.5 µm deep wells of 46 fL volume [18]. The bundles were washed several times with EDTA and ethanol followed by plasma treatment for 60 sec in Expanded plasma cleaner (Harrick Plasma, Ithaca, NY). The surface of the wells was silanized for 30 min with 150 µL silane (Gelest Inc, Morrisville, PA) in 15 mL of ethanol. The silanized fiber bundles were washed with ethanol and dried under low pressure for 1 hour. The resulting fiber bundles were placed in a nitrogen-filled container at 80°C for 30 min. The fiber bundles were stored under nitrogen in a dessicator and used within two days. Room temperature single molecule experiments on modified fibers were compared with non-modified fibers and fibers treated with BSA. No difference in activity was observed between BSA-treated and silane-treated fibers and the average activity was comparable to the bulk activity. However, since the variation in BSA passivation properties upon heating are unknown, only silane-passivated fibers were used. Additionally, the sealing proficiency of modified fibers was confirmed by photobleaching experiments. A solution of 10 µM resorufin in deionized (DI) water was enclosed in the individual wells. High intensity light was used to illuminate a small portion of the array for 20 min to photobleach resorufin. The full view image was taken and no significant leakage was observed. An additional image was taken after 15 min to confirm the integrity of seal (Figure S1B, C, D in File S1).

### Microscope/stage description

An upright Olympus B64 microscope with an attached short arc mercury lamp as an excitation light source was used. The excitation source and fluorescence emission are filtered through a filter cube (Ex. 577/10×, Em. 620/60 m, dichroic 585LP) from Chroma Technology Corp, Bellows Falls, VT. The emission light was detected by a CCD camera (Sensicam QE, Cooke Corporation, Romulus, MI) and captured images were analyzed using IPLab software (BD Bioscience, Rockville, MD). The setup used to seal the assay solution within fiber wells is described elsewhere [18] and is shown in Figure S1A in File S1. In addition to the previous setup, a flat Peltier heating plate with an attached thermocouple was installed between the heat sink and the PDMS sheet. The Peltier plate and thermocouple are controlled and recorded by a computer via LabView (National Instruments, Austin, TX). The heating plate was calibrated prior to each experiment. The temperature variations within a single experiment did not exceed more than 0.5°C. By changing the current (3.0 A), voltage (4.0 V) and pulse duration (3 sec) it was possible to heat a sample to 47°C in 4 sec and cool it back to 20°C within 20 sec (Figure S1E in File S1).

### Image processing, normalization, and data analysis

Twenty µL of 3.6 pM enzyme solution and 100 µM of RBG substrate were placed on the PDMS sheet on the heating Peltier plate. A fiber bundle containing 50 000 wells was sealed against the heating Peltier plate to create 46 fL reaction vessels. It takes approximately 30 sec. from the onset of mixing an enzyme with the substrate to the time we acquire the first image. Each individual well contains 1 or 0 enzyme molecule with the ratio of a single enzyme molecule per 10 empty wells [27]. (Figure 1A) Moreover the probability of well containing more than one molecule is around 0.45%. To obtain initial enzyme activities, six images were taken 20 seconds apart and the fluorescence signal was obtained for each well containing an active enzyme molecule. (Figure 1B) Next, five heating pulses were introduced and six images were taken between pulses. Only five images were analyzed–the first image after heating was not considered for data analysis to ensure that the system had returned to ambient temperature. The raw fluorescence intensities of the trajectories were multiplied by a calibration factor that converted fluorescence into concentration and were background correlated (Table S1 in File S2). The substrate turnover was calculated for each enzyme before and between heating pulses using the equation S(t) = F′(t)+k_ph_×F(t), where S(t) is substrate turnover in sec^−1^, F(t) is fluorescence intensity as a function of time, F′(t) is the fluorescence intensity time derivative and k_ph_ is a photobleaching rate [18]. (Table S1, S2 in File S2) The photobleaching rate was determined by trapping 10 µM of fluorescent product and monitoring the fluorescence decreases every 20 sec with an exposure of 2 sec over 1 hour.

## Conclusion

In this paper, we developed a platform capable of observing the activities of thousands of single enzyme molecules and perturbing them using thermal energy. Using this platform, we observed that individual β-galactosidase molecules have numerous stable activity states that can be interconverted by providing thermal energy to the system. This work proves that these activity changes are the result of conformational changes, however it does not eliminate the possibility that sequence differences between molecules may also lead to the heterogeneity in activity. The ability to perturb enzyme conformation using thermal energy provides a new tool for studying single enzyme molecules that can lead to new insights into fundamental biochemistry.

## Supporting Information

File S1 (DOCX)

File S2 (DOCX)
