# Supplementary material for: Observing Single Enzyme Molecules Interconvert between Activity States upon Heating
Source: PLoS One. 2014 Jan 21;9(1):e86224. doi: 10.1371/journal.pone.0086224 (PMC3897666; doi:10.1371/journal.pone.0086224)
Supplement: File S1 — (DOCX) [file pone.0086224.s001.docx]

**Supporting Information**


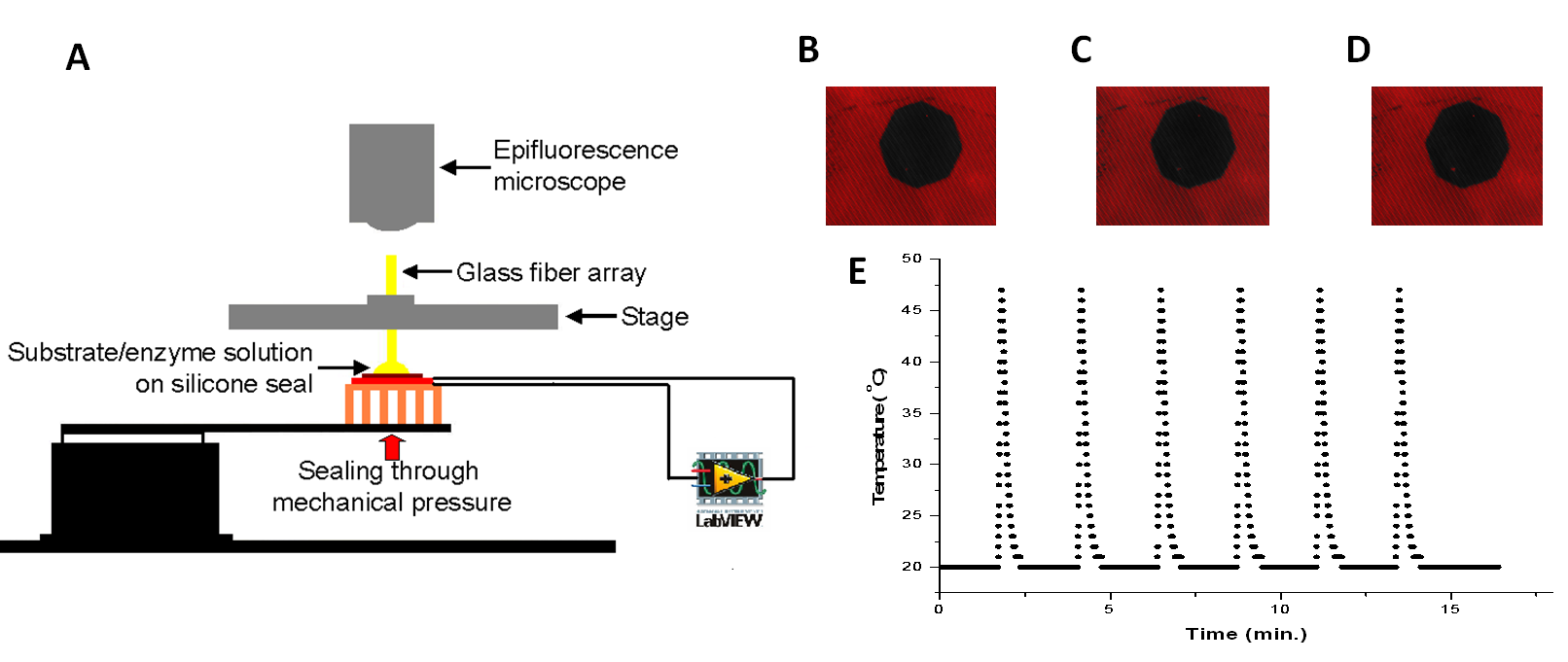


**Figure S1. The experimental platform for studying changes in the kinetics of single non-immobilized enzyme molecules.**

(A) Schematic of the custom-made microscope stage with installed Peltier plate controlled by LabView. (B) Fluorescent images taken after photobleaching of 10 μM resorufin, (C) after introducing six heating pulses, and (D) 15 min after the pulses shows the seal remains intact. (E) The plot of temperature pulses over the time span recorded by a thermocouple sensor.


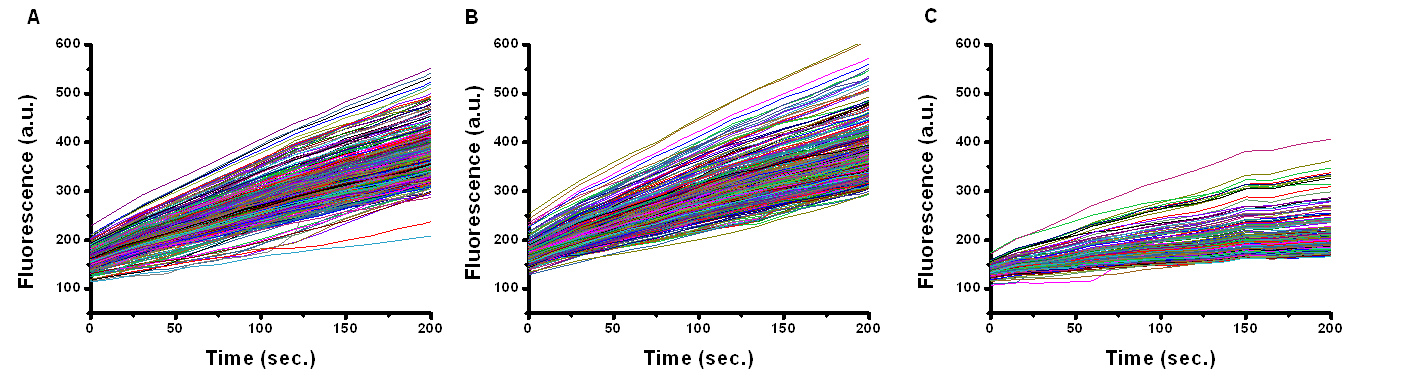


**Figure S2. Activity traces of single enzymes for different surface modifications of the fiber.**

A and B represent modifications with BSA blocking buffer and silane respectively, C is an un-modified fiber.


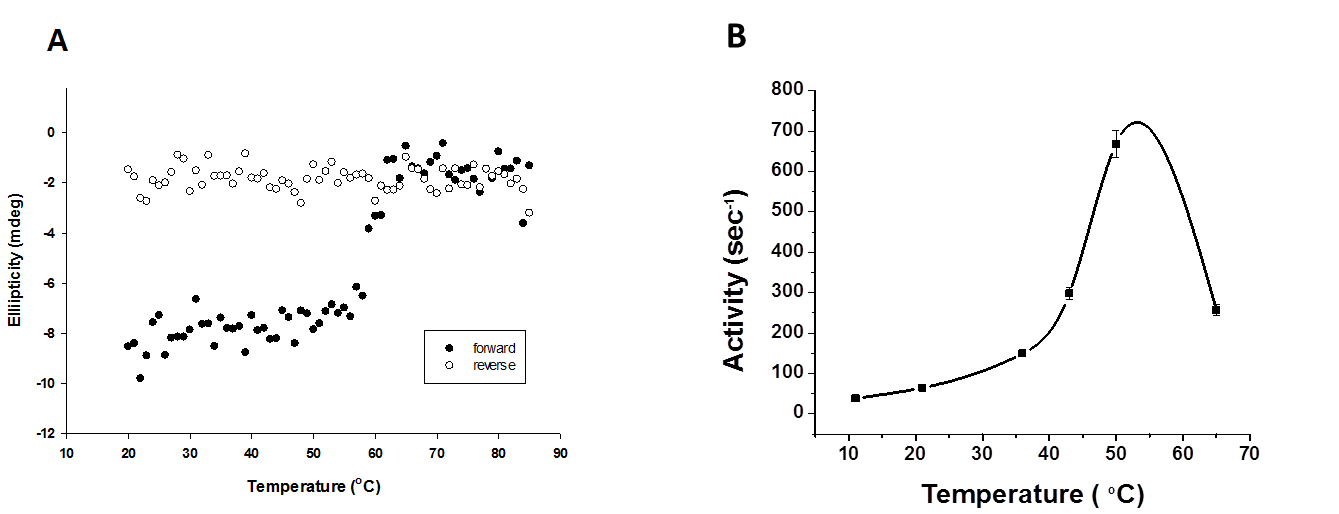


Figure S3. Thermal denaturation of β-galactosidase.

(A) Circular dichroism spectrum of the enzyme sample as a function of temperature. The experiment was performed on a JASCO J-720 spectropolarimeter with an attached Peltier pump. The readings were obtained at 222nm with a temperature ramp of 1°C per minute. Both forward and reverse temperature measurements were acquired. (B) Change in the activity of the enzyme molecules with respect to temperature. The heating stage was equilibrated to a specific temperature and the activities of the trapped enzymes were obtained. Each temperature point corresponds to the average activity of about 1000 enzymes.

**Figure S4. First order relationship between heating pulses and natural logarithm of the number of denatured molecules.**

A denatured molecule is defined as a molecule that does not show any activity after a heating pulse. Molecules that are not active but gain activity after a heating pulse are not considered denatured. The calculated rate of denaturation is equal to 0.6% of the total population per heating pulse with an R^2^ value of 0.98.

**
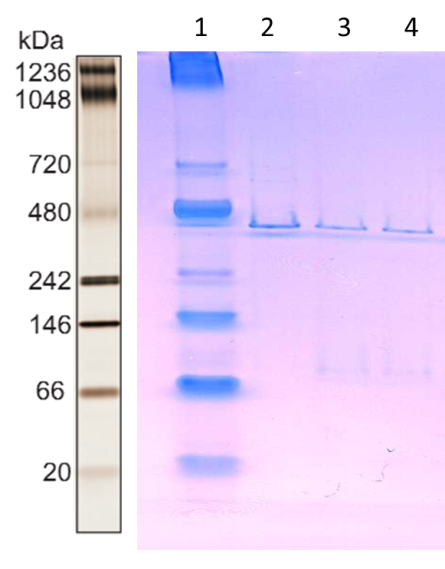
**

Figure S5. Native Novex® Tris-Glycine polyacrylamide gel of β-galactosidase.

Lane 1 is a ladder, lane 2 is purified β-galactosidase, lanes 3 and 4 are enzymes heated at 47 ^°^C for 2 min and 4 min.

**Figure S6. Normalized average activities of the enzymes.**

The enzymatic assay was performed with unheated substrate and substrate that was exposed to different temperatures (40 °C, 45 °C, 50 °C) for 1 min. and then cooled to room temperature. The activity was normalized against the unheated substrate assay. Error bars correspond to three different experiments. The blank experiments where only substrate was present showed no change in fluorescence.

**Figure S7. Normalized average product fluorescence intensities.**

100 µM of fluorescent product was heated at different temperatures (40 °C, 45 °C, 50 °C) for 1 min and cooled to room temperature. The fluorescence intensities were measured every 30 sec. over 10 min. The results were averaged and normalized against the fluorescence of unheated product. Error bars correspond to three different measurements of the fluorescent intensities.
