# Supplementary material for: Observing Single Enzyme Molecules Interconvert between Activity States upon Heating
Source: PLoS One. 2014 Jan 21;9(1):e86224. doi: 10.1371/journal.pone.0086224 (PMC3897666; doi:10.1371/journal.pone.0086224)
Supplement: File S2 — (DOCX) [file pone.0086224.s002.docx]

Table S1. Calculated number of fluorescent product molecules per active well for two randomly chosen enzymes.

The raw fluorescence intensities were obtained from individual wells (average intensity of 81 pixels per well). Intensities are plotted with respect to time. The intensity of 50 non-active wells per fiber were collected and averaged as a background signal. The active enzyme intensities were corrected with respect to background and multiplied by a standard factor of 320 (molecules/FU). The standard factor was obtained from a standard curve generated using known concentrations of fluorescent product. The data highlighted in yellow are not included in the analysis as they were collected directly after the heat pulse and before the temperature had settled.

Table S2. Linear regression analysis for enzymes I and II for all periods between the pulses.

The number of product molecules were plotted against time and fitted using a linear regression tool. Each slope F’(t) is corrected for photobleaching. The final rate S(t) is calculated from the equation S(t) = F’(t) + F(t) x k_ph_.
